# Differences in risk-factor profiles between patients with ESBL-producing *Escherichia coli* and *Klebsiella pneumoniae*: a multicentre case-case comparison study

**DOI:** 10.1186/2047-2994-3-27

**Published:** 2014-09-01

**Authors:** Joshua T Freeman, Joseph Rubin, Gary N McAuliffe, Gisele Peirano, Sally A Roberts, Dragana Drinković, Johann DD Pitout

**Affiliations:** 1 Department of Clinical Microbiology, Auckland District Health Board, Auckland City Hospital, Auckland, New Zealand; 2 Faculty of Medical and Health Sciences, University of Auckland, Auckland, New Zealand; 3 Division of Microbiology, Calgary Laboratory Services, Departments of Pathology & Laboratory Medicine, Microbiology Immunology and Infectious Diseases, University of Calgary, Calgary, Alberta, Canada; 4 Department of Clinical Microbiology, Waitemata District Health Board, Auckland, New Zealand

**Keywords:** ESBL, *Klebsiella pneumoniae*, *Escherichia coli*, Bacteraemia, Risk factors

## Abstract

**Background:**

Generic epidemiological differences between extended-spectrum beta-lactamase (ESBL)-producing *Escherichia coli* (ESBL-EC) and *Klebsiella pneumoniae* (ESBL-KP), are poorly defined. Nonetheless, defining such differences and understanding their basis could have strategic implications for infection control policy and practice.

**Methods:**

Between 2009 and 2011 patients with bacteraemia due to ESBL-EC or ESBL-KP across all three acute hospitals in the city of Auckland, New Zealand, were eligible for inclusion. Recognised risk factors for ESBL bacteraemia were compared between species in a retrospective case-case study design using multivariate logistic regression. Representative isolates underwent ESBL gene characterisation and molecular typing.

**Results:**

170 patients and 176 isolates were included in the study (92 patients with ESBL-EC*,* 78 with ESBL-KP). 92.6% had CTX-Ms. 39% of EC were ST131 while 51% of KP belonged to 3 different STs (i.e. ST20, ST48 & ST1087). Specific sequence types were associated with specific hospitals for ESBL-KP but not ESBL-EC. Variables positively associated with ESBL-EC on multivariate analysis were: community acquired infection (odds ratio [OR] 7.9; 95% CI: 2.6-23.9); chronic pulmonary disease (OR 5.5; 95% CI: 1.5-20.1); and high prevalence country of origin (OR 4.3; 95% CI: 1.6-11.6). Variables negatively associated with ESBL-EC were previous transplant (OR 0.06; 95% CI: 0.007-0.6); Hospital 2 (OR 0.3; 95% CI: 0.1-0.7) and recent ICU admission (OR 0.3; 95% CI: 0.07-0.9).

**Conclusions:**

Differences in risk profiles between patients with ESBL-EC and ESBL-KP suggest fundamental differences in transmission dynamics. Understanding the biological basis for these differences could have implications for infection control practice. Tailoring of infection control measures according to ESBL species may be indicated in some instances.

## Background

The global dissemination of extended-spectrum β-lactamase producing Enterobacteriaceae (ESBL-E) poses a growing challenge to both public health and hospital infection control services [1].Whereas early reports of ESBL-E typically described hospital outbreaks of TEM and SHV producing *Klebsiella pneumoniae,* over the last decade reports have focused on CTX-M, which has become the overwhelmingly predominant ESBL subclass worldwide [2]. The rise of CTX-M has been characterized by increased rates of community transmission and an association with *Escherichia coli*. More recently however, large epidemics of CTX-M producing *K. pneumoniae* have also been reported [3-5]. Despite this development, few studies to identify risk factors for ESBL-E have focused specifically on *K. pneumoniae.* Although several studies have combined different ESBL-E species together into single case groups [6,7] most have focused exclusively on CTX-M producing *E. coli*[8-10]*.* Moreover, while epidemiological differences between these species have been assumed in recent guidelines [11], few studies have sought to systematically characterize and quantify these differences. Nonetheless, defining such differences and better understanding their biological basis could have strategic implications for policy and practice; both for community public health and hospital infection control.

In view of this background, we sought to systematically identify and quantify differences in the risk factor profiles between patients with ESBL-*E. coli* (ESBL-EC) and ESBL-*K. pneumoniae* (ESBL-KP) bacteraemia over a three year period.

## Methods

### Study design/inclusion criteria

We performed a retrospective case-case study of patients admitted to hospital with ESBL-E bacteraemia in the Auckland region of New Zealand over a 3 year period in order to identify and quantify differences in the risk factor profiles of patients with ESBL-EC and ESBL-KP bacteraemia. A 3 year period was chosen because it was estimated that this would provide over 70 patients in both the ESBL-EC and ESBL-KP groups, a number which we speculated would allow for meaningful statistical comparison between the two groups. The absence of similar previous case-case comparisons precluded a formal power calculation. All patients with blood culture isolates of either ESBL-EC or ESBL-KP in Auckland, New Zealand, between January 1^st^ 2009 and December 31st 2011 were eligible for inclusion. All 3 public hospitals providing acute medical care to the Auckland population of approximately 1.4 million participated in the study (in the Auckland region, all acute medical care is provided by public hospitals). These 3 hospitals provide a mix of tertiary and secondary care under medical and surgical specialties and subspecialties, ranging in approximate size from 600–900 beds (Hospital 1–900 beds [700 adult and 200 paediatric]; Hospital 2–800 beds [725 adult and 75 paediatric] and Hospital 3–600 beds [590 adult and 10 paediatric]). For the duration of the study, laboratories at all 3 hospitals had a policy of routinely stocking all isolates of ESBL-E from blood at −70°C. Representative isolates were included for each patient bacteraemia episode. For patients with multiple bacteraemia episodes with the same species, only the first episode was included. Patients with both ESBL-EC and ESBL-KP simultaneously were excluded from the risk factor analysis as were patients that had successive bacteraemia episodes with each of the two species. Patients with no available isolate were also excluded. Both paediatric and adult patients were eligible for inclusion. Approval was obtained from the Northern Regional Ethics Committee.

### Patient data collection

Data were collected from the electronic medical records by a single investigator. Because the hospitals involved in the study provide healthcare to the entire Auckland region, complete and reliable data on the timing and number of admissions to hospital in the Auckland area were available for all patients. Patient risk factors were broadly divided into: 1) factors relating to acquisition (including demographic characteristics), 2) source of bacteraemia, 3) comorbidities.

Community-onset infection was defined as a positive blood culture collected within the first 48 hours of hospital admission. Community acquisition was defined according to a modified version of the Friedman criteria [12] and was ascribed to patients having all 4 of the following characteristics: 1) no hospital admissions in the Auckland region during the preceding year, 2) not a long term care facility resident, 3) not a dialysis patient, 4) community-onset infection. High prevalence regions were defined as China, India, Pakistan, South America, Africa, the Middle East and Southeast Asia [13,14]. Country of origin was assigned on the basis of self reported ethnicity/country of origin data recorded routinely on admission to hospitals in the Auckland region. The designation of New Zealand Maori or NZ European ethnicity was also made on the basis of this data. Recent ICU admission was defined as patients who had been admitted to ICU within the last 3 months, excluding the current admission. The source of bacteraemia was determined through an assessment of the clinical records and microbiology findings. Febrile neutropaenia was defined as bacteraemia with no clear primary source in patients with fever and a neutrophil count of less than 0.5 × 10^9^/L. Gastrointestinal infections included intra-abdominal gastrointestinal infections as well as infection due to acute hepatobiliary obstruction. Central line infections were defined as bacteraemia in non-neutropaenic patients with a central line and no clear alternative primary source. Prostate biopsy related infections were distinguished from other UTI by a history of a trans-rectal prostate biopsy in the preceding 72 hours. Recurrent UTIs were defined as a record of two or more previous admissions to hospital with UTI or documented evidence of recurrent UTI in the community. Patients were defined as having a long term catheter if either a urethral or suprapubic catheter was present on admission to hospital. Transplants included both hematopoietic stem cell and solid organ. Chronic pulmonary disease required documented evidence of asthma, chronic obstructive airways disease or other chronic respiratory diseases in the clinical records. Other definitions were defined as per Charlson et al. [15].

### Microbiology and molecular testing of isolates

Blood culture isolates of ESBL-E across all 3 laboratories in the Auckland area are routinely frozen and stocked in skim milk or other suspension medium at −70°C. Stocked isolates were sub-cultured onto nutrient agar slopes prior to molecular analysis. Susceptibility testing and ESBL screening and confirmation tests were performed according to the 2009 CLSI guidelines [16]. Isoelectric focusing (IEF) was performed on freeze-thaw extracts as previously described [17]. Polymerase chain reaction (PCR) amplification and sequencing for *bla*_CTX-Ms_, *bla*_OXAs_, *bla*_TEMs_, *bla*_SHV_, was carried out on the isolates with a GeneAmp 9700 ThermoCycler instrument (Applied Biosystems, Norwalk, Con) using PCR conditions and primers as previously described [17]. Additional investigations included PCR for plasmid-mediated quinolone resistance determinants *aac-(6’)-Ib-cr*, *qnr B*, *qnrA* and *qnrS*[17].

Genetic relatedness was examined by pulsed-field gel electrophoresis (PFGE) following extraction of genomic DNA and digestion with *Xba*I using the *E. coli* (O157:H7) protocol established by the Centers for Disease Control and Prevention, Atlanta, GA [18]. Cluster designation was based on isolates showing approximately 80% or greater relatedness, which corresponds to the “possibly related (4–6 bands difference)” criteria of Tenover et al. [19]. Multilocus sequence typing (MLST) was performed using standard protocols for *E. coli* (http://mlst.warwick.ac.uk/mlst/dbs/Ecoli) and *K. pneumoniae* (http://www.pasteur.fr/recherche/genopole/PF8/mlst/Kpneumoniae.html). MLST was performed on pulsed field clusters consisting of ≥10 isolates.

### Statistical analysis

The primary analysis involved using ESBL-EC bacteraemia as the dependent variable and using patients with ESBL-KP as a comparator group. For each potential risk factor, odds ratios and 95% confidence intervals were calculated using univariable logistic regression. Chi square test or Fisher’s exact test and Wilcoxon rank sum tests were used to compare categorical and continuous variables respectively. Age, gender and hospital were considered eligible for inclusion in a multivariate analysis on an a priori basis along with demographic and comorbidity risk factor variables with a p value of less than 0.1. Underlying sources of bacteraemia and the timing of infection (ie: community or hospital onset) were excluded from the multivariate analysis because they occurred concurrently with the outcome and were therefore not considered to be risk factors in the conventional sense. The final model was derived using forward logistic regression. All statistical testing was two tailed and statistical significance was defined as P ≤ 0.05. Statistical analyses were performed using SPSS software (Version 21.0).

## Results

Across all 3 sites during the study period there were a total of 206 patients with bloodstream infections with ESBL-EC and ESBL-KP (89, 57, and 60 from hospitals 1, 2 and 3 respectively). Of these, 173 patients (84.0%) had isolates available and were included in the study (33 patients were excluded due to not having an isolate available). These 173 patients had a total of 176 isolates because two patients had simultaneous bacteraemia with ESBL-EC and ESBL-KP and one patient had successive bacteraemic episodes, firstly with ESBL-KP and then ESBL-EC over a year later. These 3 patients were excluded from the risk factor analysis. This left 170 patients for the risk factor analysis [92 patients with ESBL-EC (54%) and 78 with ESBL-KP (46%)]. The percentage of isolates available from each hospital were 84/90 (93.3%) from Hospital 1; 57/59 (96.6%) from Hospital 2 and 35/60 (58.3%) from Hospital 3. The proportion with ESBL-EC among available isolates in each hospital was 64.3% for Hospital 1; 40.4% for Hospital 2; and 51.4% for Hospital 3. Among excluded patients with no available isolate, the proportion with ESBL-EC was 51%.

Overall CTX-M ESBLs accounted for 92.6% of the 176 isolates. CTX-M-15 predominated (72.7%). Of the ESBL-EC, 29% produced CTX-M-14, 61% CTX-M-15 and 4% TEM-52, while 1 each produced CTX-M-24, CTX-M-27 and SHV-12. Overall 39% belonged to ST131. Of the 81 ESBL-KP, 2% produced CTX-M-14, 86% CTX-M-15, 5% SHV2a while 1 each produced SHV-12 and TEM-52. ESBL-KP isolates were more likely than ESBL-EC to carry fluoroquinolone resistance genes *aac-(6’)-Ib-cr* and *qnrB* in combination (68% versus 10% respectively) but were less likely to carry CTX-M-14 and TEM-1 (Table 1). ESBL-KP had significantly higher rates of resistance to gentamicin and trimethoprim-sulfamethoxazole as well as higher rates of resistance to 4 or more antibiotic classes (Table 1). Rates of fluoroquinolone resistance were similar.

**Table 1:** Comparison of ESBL-EC and ESBL-KP with respect to molecular characteristics and susceptibility profiles

	**ESBL-EC (%)**	**ESBL-KP (%)**	**P value**
*aac(6’)-Ib-cr*	41/95 (43.1)	61/81 (75.3)	<0.0001
*qnrB*	11/95 (11.6)	65/81 (80.2)	<0.0001
*bla*_CTX-M-15_	58/95 (61.1)	70/81 (86.4)	0.0002
*bla*_CTX-M-14_	28/95 (29.5)	2/81 (2.5)	<0.0001
*bla*_TEM-1_	53/95 (55.8)	20/81 (24.7)	<0.0001
*bla*_OXA_	42/95 (44.2)	50/81 (61.7)	0.024
Other *bla*_CTX-M_	4/95 (4.2)	1/81 (1.2)	0.38
Non *bla*_CTX-M_ ESBLs	5/95 (5.3)	8/81 (9.9)	0.26
Gentamicin NS	53/95 (55.8)	66/81 (81.5)	0.0003
Trimethoprim-sulfamethoxazole NS	67/95 (70.5)	78/81 (96.3)	<0.0001
Fluoroquinolone NS	66/95 (69.5)	62/81 (76.5)	0.31
NS ≥ 4 antibiotic classes	36/95 (37.9)	55/81 (67.9)	<0.0001

NS, non-susceptible. Representative antibiotics for the three non-beta-lactam antibiotic classes tested were: gentamicin; trimethoprim-sulfamethoxazole, ciprofloxacin. All isolates were assumed by definition to be resistant to cephalosporins.

PFGE identified 3 major clusters (i.e. more than 10 isolates per cluster) among ESBL-KP that were identified by MLST as: ST20 (n = 15); ST48 (n = 13) and ST1087 (n = 13). ST1087 is a novel ST that produced CTX-M-15 in combination with OXA-1 and TEM-1 as well as *aac(6)-lb-cr* and *qnrB.* It was also noted to be less susceptible to ciprofloxacin (0/13 vs 18/68; p = 0.03) and to cause higher rates of infection in febrile neutropaenic patients than other ESBL-KP isolates (5/13 vs 7/68; p = 0.02). When community acquired isolates were excluded, there was a significant association between ST48 and Hospital 3 (7/15 vs 6/60; p = 0.003) as well as ST1087 and Hospital 2 (10/32 vs 3/43; p = 0.01). ST20 was also more common among patients from Hospital 2 although this was not statistically significant (8/32 vs 5/43; p = 0.19). PFGE identified 1 major cluster (i.e. 37/95 [38.9%]) among the ESBL-EC that was identified as ST131. No significant associations were observed between ST131 and any particular hospital.

Characteristics associated with ESBL-EC bloodstream infection on univariable analysis included having a high prevalence country of origin (Table 2). Patients with ESBL-EC were also more likely to have community acquired disease or chronic pulmonary disease, but were less likely to have been recently admitted to ICU or Hospital 2 and were less likely to have had a previous transplant. For ESBL-EC, the underlying source of bacteraemia was more likely to be related to prostate biopsy but less likely to be associated with febrile neutropaenia or central line infection. Several of these associations remained significant on multivariate analysis (Table 2). Because only 51% of eligible cases were included from Hospital 3, the multivariate analysis was repeated with Hospital 3 cases excluded in order to evaluate the possibility that incomplete data may biased our findings. When taking this approach, all variables found to be significant in the primary analysis remained significant with the exception of previous transplant (data not shown).

**Table 2:** Adjusted and unadjusted odds ratios with ESBL-EC bacteraemia as the dependent variable

	**ESBL-EC (%)**	**ESBL-KP (%)**	**Crude OR (95% CI)**^**d**^**/P value**	**Adjusted OR (95% CI)**^**d**^
**Demographics/acquisition**				
Male gender^a^	60/92 (65.2)	49/78 (62.8)	1.1 (0.6–2.2)	
Median age (IQR)^a^	65.5 (53–76)	61.5 (38.5-75.5)	P = 0.01	
High prevalence country^a^	35/92 (38.0)	8/78 (10.3)	5.4 (2.2–13.7)	4.3 (1.6–11.6)
Pacific Islander	10/92 (10.9)	14/78 (17.9)	0.6 (0.2–1.4)	
NZ Maori	2/92 (2.2)	6/78 (7.7)	0.3 (0.04–1.5)	
LTCF residence	11/92 (12.0)	8/78 (10.3)	1.2 (0.4–3.5)	
Community acquisition^a^	35/92 (38.0)	6/78 (7.7)	7.4 (2.7–21.1)	7.9 (2.6–23.9)
Community onset infection	73/92 (79.3)	37/78 (47.4)	4.3 (2.1–8.8)	
Recent ICU admission^a^	5/92 (5.4)	14/78 (17.9)	0.3 (0.08–0.8)	
Median hospital days (IQR)	1.5 (0–16)	9.5 (0–30.5)	P <0.0001	
Hospital 1^a^	53/92 (57.6)	29/78 (37.1)	2.3 (1.2–4.5)	
Hospital 2^a^	21/92 (22.8)	32/78 (41.0)	0.4 (0.2–0.9)	0.3 (0.1–0.7)
Hospital 3^a^	18/92 (19.6)	17/78 (21.8)	0.9 (0.4–2.0)	
**Source of bacteraemia**				
Urinary tract	49/92 (53.3)	33/78 (42.3)	1.6 (0.8–3.0)	
Gastrointestinal	17/92 (18.5)	10/78 (12.8)	1.5 (0.6–3.9)	
Central line	0/92 (1.1)	5/78 (7.7)	P = 0.02	
Febrile neutropenia	6/92 (5.4)	13/78 (15.4)	0.3 (0.1–1.1)	
Prostate biopsy related	8/92 (8.7)	0/78 (0)	P = 0.008	
Bone and joint	0/92 (0)	3/78 (3.8)	P = 0.095	
Respiratory tract	6/92(6.5)	3/78 (3.8)	1.7 (0.4–9.2)	
Uncertain^c^	4/92 (4.3)	9/78 (11.5)	0.3 (0.1–1.3)	
**Comorbidities**				
Diabetes	22/92 (23.9)	14/78 (17.9)	1.4 (0.6–3.3)	
Recurrent UTI	4/92 (4.3)	5/78 (6.4)	0.7 (0.1–3.0)	
Long term IDC	11/92 (12.0)	12/78 (15.4)	0.7 (0.3–2.0)	
Solid tumour	17/92 (18.5)	13/78 (16.7)	1.1 (0.5–2.7)	
Haematological malignancy	12/92 (13.0)	16/78 (20.5)	0.6 (0.2–1.4)	
Previous transplant^a,b^	1/92 (1.1)	7/78 (9.0)	0.1 (0.005–0.9)	0.06 (0.007–0.6)
Dialysis	4/92 (4.3)	2/78 (2.6)	1.7 (0.3–14.0)	
CPD^a^	14/92 (15.2)	4/78 (5.1)	3.3 (1.0–12.6)	5.5 (1.5–20.1)
Median number comorbidities (IQR)	1 (1–3)	1 (1–2)	P = 0.39	

^a^Indicates that variable was included in the multivariate analysis.

^b^4 patients with liver transplants, and 4 patients with hematopoietic stem cell transplants transplants (one additional patient with a liver transplant had separate episodes of ESBL-EC and ESBL-KP bacteremia).

^c^Uncertain sources were those where the treating clinician was unable determine the aetiology.

^d^P values are included in this column for continuous variables and dichotomous variables for which an OR could not be calculated.

IQR, interquartile range; LTCF, long term care facility; UTI, urinary tract infection; IDC, Indwelling urinary catheter; CPD, Chronic pulmonary disease.

## Discussion

Our findings demonstrate that even when CTX-M is predominant in both species, patients with bacteraemia due to *E. coli* and *K. pneumoniae* have substantially different risk profiles. In addition, several findings suggest that these differences are explained by underlying differences in transmission dynamics between species. Firstly, the higher rate of community acquisition among ESBL-EC indicates that community transmission plays a more important role for ESBL-EC than ESBL-KP. This notion is supported by the observation that ESBL-EC had more diverse pulsed field patterns and resistance gene profiles, consistent with more distant epidemiological linkage between isolates. Previous surveys describe similar differences in clonal diversity between these two species [20,21]. Furthermore, we observed no association between any particular hospital and ST131 whereas ESBL-KP in general was associated with Hospital 2 and specific hospitals were associated with specific sequence types, including ST-48. Of note, ST-48 is a widely disseminated international clone that has been described in the literature as a cause of hospital outbreaks [22,23].

Secondly, we found patients with ESBL-EC were more likely to come from high prevalence countries. Although country of origin has been highlighted previously as a risk factor for ESBL-EC compared to susceptible *E. coli*, our findings suggest that having a high prevalence country of origin is more strongly associated with ESBL-EC than for ESBL-KP. Possible explanations for this include higher rates of acquisition of ESBL-EC during travel to high prevalence regions, either by the patients themselves or household members [24,25]. Either way, this observation further supports the notion that ESBL-EC is more likely than ESBL-KP to be acquired in community settings.

In contrast, we found that ESBL-KP was associated with recent ICU admission and previous transplantation which may be explained by ESBL-KP having higher rates of transmission in the ICU or transplant ward setting [26,27]. Several other recent studies also found that ESBL-KP has higher rates of transmission in healthcare settings, suggesting that the inter-species differences in epidemiology we describe are not unique to our region [26-29]. The reason for these inter-species differences in transmissibility is not well understood although recent work suggests that differences in rates of environmental contamination with viable organisms may be part of the explanation [30].

We also found that while the overall range of infections caused by the two species was similar, ESBL-KP was associated with febrile neutropenia and central line infections whereas ESBL-EC was associated with infections following prostatic biopsy. The explanation for these observations are uncertain but contributing factors may also include underlying differences in transmission dynamics since patients with febrile neutropaenia and central line infections typically have more previous healthcare exposure than patients with bacteraemia following an outpatient prostate biopsy.

Unexpectedly, we found an independent association between ESBL-EC and chronic pulmonary disease. Similar findings have been reported previously. Another study performed in the Auckland region, comparing ESBL-EC to susceptible *E. coli*, also found an independent association between ESBL-EC and chronic obstructive pulmonary disease [31]. Another large multicentre Spanish study also found chronic obstructive pulmonary disease to be an independent risk factor for ESBL-EC [32]. Further investigation is necessary to determine whether this observation is due to a type 1 error, unmeasured confounding factors or underlying causal mechanisms that are yet to be characterized.

This is the first report of *K. pneumoniae* ST1087 which we found to be associated with high rates of resistance to ciprofloxacin, Hospital 2 and high rates of bacteraemia among neutropaenic patients. More work is needed to determine the geographic distribution of this clone and its importance as a healthcare-associated pathogen.

Limitations of our study include retrospective data collection and lack of information on antibiotic exposure and travel history as well as the exclusion of a number of patients with no isolate available. Further limitations include the case-case study design, which does not allow for identification of risk factors common to both organisms; the inclusion of bacteraemic patients only, which limits the applicability of our findings to other infections; and the use of PFGE and MLST which compared to whole genome sequencing, provide less information about the degree of genetic relatedness between isolates. Strengths of the study include the inclusion of data from all acute care hospitals in the region; the availability of comprehensive and dependable data on previous hospital admissions in the region; the molecular characterization of all isolates, and the current lack of published data describing epidemiological differences between ESBL-EC and ESBL-KP. Our findings also suggest that studies investigating risk factors for ESBL-E may fail to detect relevant associations if both species are combined as a single “case group”. Moreover, recognized risk factors should not be assumed to apply equally to both species. As studies are performed on other important emerging resistance mechanisms in Enterobacteriaceae such as NDM and KPC, these methodological considerations are worth noting.

## Conclusion

Our findings demonstrate that even when CTX-M is predominant in both species, the epidemiological risk profiles of patients with ESBL-EC and ESBL-KP are substantially different. Although further studies are needed, this seems to be largely explained by differences between the two species in terms of their propensity to transmit in community and healthcare settings. Further work is needed to determine the biological basis for these differences. As further insights are gained, new opportunities will arise to expand and refine our repertoire of strategies to prevent transmission of these organisms in both community and healthcare settings. In the interim, our findings imply that a single set of hospital infection control measures is unlikely to be equally effective against both species and that tailored approaches to infection control according to ESBL species may be helpful in some instances.

## Competing interests

JDDP had previously received research funds from Merck and Astra Zeneca. GP, JR GM & DD nothing to declare. JTF has previously received a speaking honorarium and a conference airfare paid by MSD and a conference airfare paid by Pfizer. This work was supported by an investigator initiated study grant from Merck Sharp and Dohme (IISP 40321). This work was also supported by a research grant from the Calgary Laboratory Services (# 73–6350) and was presented at the 53rd ICAAC in Denver, CO, USA (abstract no C2-1112).

## Authors’ contributions

JTF initiated the design of the study, provided oversight of data collection, performed the statistical analyses and wrote the manuscript. GNM contributed to the design of the study, collected epidemiological data from the patient records and contributed to review of the manuscript. DD and SAR contributed to the initial design of the study, data collection, recovery of isolates and review of the final manuscript. JR, GP performed molecular testing for ESBL and PMQR genes as well as MLST and PFGE. JDDP contributed to the design of the study, oversight of molecular testing and review of the final manuscript. All authors read and approved the final manuscript.

## References

[B1] Pitout JD Laupland KB Extended-spectrum beta-lactamase-producing Enterobacteriaceae: an emerging public health concern Lancet Infect Dis 2008 8 159 166 10.1016/S1473-3099(08)70041-0 18291338

[B2] Cantón R Coque TM The CTX-M β-lactamase pandemic Curr Opin Microbiol 2006 9 466 475 10.1016/j.mib.2006.08.011 16942899

[B3] Damjanova I Tóth A Pászti J Hajbel-Vékony G Jakab M Berta J Milch H Füzi M Expansion and countrywide dissemination of ST11, ST15 and ST147 ciprofloxacin-resistant CTX-M-15-type beta-lactamase-producing Klebsiella pneumoniae epidemic clones in Hungary in 2005–the new ‘MRSAs’? J Antimicrob Chemother 2008 62 978 985 10.1093/jac/dkn287 18667450

[B4] Coelho A Mirelis B Alonso-Tarres C Nieves Larrosa M Miró E Clivillé Abad R Bartolomé RM Castañer M Prats G Johnson JR Navarro F González-López JJ Detection of three stable genetic clones of CTX-M-15-producing *Klebsiella pneumoniae* in the Barcelona metropolitan area, Spain J Antimicrob Chemother 2009 64 862 864 10.1093/jac/dkp264 19628471

[B5] Pierano G Hung King Sang J Pitondo-silva A Laupland KB Pitout JD Molecular epidemiology of extended-spectrum beta-lactamase-producing *Klebsiella pneumoniae* over a 10 year period in Calgary, Canada J Antimicrob Chemother 2012 67 1114 1120 10.1093/jac/dks026 22334607

[B6] Park SY Kang CI Joo EJ Ha YE Wi YM Chung DR Peck KR Lee NY Song JH Risk factors for multidrug resistance in nosocomial bacteremia caused by extended-spectrum β-lactamase-producing *Escherichia coli* and *Klebsiella pneumoniae* Microb Drug Resist 2012 18 518 524 10.1089/mdr.2012.0067 22742454

[B7] Freeman JT McBride SJ Nisbet MS Gamble GD Williamson DA Taylor SL Holland DJ Bloodstream infection with extended-spectrum beta-lactamase-producing Enterobacteriaceae at a tertiary care hospital in New Zealand: risk factors and outcomes Int J Infect Dis 2012 16 371 374 10.1016/j.ijid.2012.01.008 22401750

[B8] Ben Ami R Schwaber MJ Nanon-Venezia S Schwartz D Giladi M Chmelnitsky I Leavitt A Carmeli Y Influx of extended-spectrum β-lactamase-producing Enterobacteriaceae into the hospital Clin Infect Dis 2006 42 925 934 10.1086/500936 16511754

[B9] Hayakawa K Gattu S Marchaim D Bhargava A Palla M Alshabani K Gudur UM Pulluru H Bathina P Sundaragiri PR Sarkar M Kakarlapudi H Ramasamy B Nanjireddy P Mohin S Dasagi M Datla S Kuchipudi V Reddy S Shahani S Upputuri V Marrey S Gannamani V Madhanagopal N Annangi S Sudha B Muppavarapu KS Moshos JA Lephart PR Pogue JM Epidemiology and risk factors for isolation of *Escherichia coli* producing CTX-M-type extended-spectrum β-lactamase in a large US medical center Antimicrob Agents Chemother 2013 57 4010 4018 10.1128/AAC.02516-12 23752516 PMC3719715

[B10] Kang CI Wi YM Lee MY Ko KS Chung DR Peck KR Lee NY Song JH Epidemiology and risk factors of community onset infections caused by extended-spectrum β-lactamase-producing *Escherichia coli* strains J Clin Microbiol 2012 50 312 317 10.1128/JCM.06002-11 22162561 PMC3264158

[B11] Tacconelli E Cataldo MA Dancer SJ De Angelis G Falcone M Frank U Kahlmeter G Pan A Petrosillo N Rodríguez-Baño J Singh N Venditti M Yokoe DS Cookson B European Society of Clinical Microbiology ESCMID guidelines for the management of the infection control measures to reduce transmission of multidrug-resistant Gram-negative bacteria in hospitalized patients Clin Microbiol Infect 2014 20 Suppl 1 1 55 24329732 10.1111/1469-0691.12427

[B12] Friedman ND Kaye KS Stout JE McGarry SA Trivette SL Briggs JP Lamm W Clark C MacFarquhar J Walton AL Reller LB Sexton DJ Health care–associated bloodstream infections in adults: a reason to change the accepted definition of community-acquired infections Ann Intern Med 2002 137 791 797 10.7326/0003-4819-137-10-200211190-00007 12435215

[B13] Hsueh PR Badal RE Hawser SP Hoban DJ Bouchillon SK Ni Y Paterson DL 2008 Asia–Pacific SMART Group Epidemiology and antimicrobial susceptibility profiles of aerobic and facultative gram negative bacilli isolated from patients with intra-abdominal infections in the Asia-Pacific region: 2008 results from SMART (Study for Monitoring Antimicrobial Resistance Trends) Int J Antimicrob Agents 2010 36 408 414 10.1016/j.ijantimicag.2010.07.002 20728316

[B14] Abdul Rahman EM El-Sherif RH High rates of intestinal colonization with extended-spectrum beta-lactamase-producing Enterobacteriaceae among healthy individuals J Investig Med 2011 59 1284 1286 22068018 10.2130/JIM.0b013e318238748e

[B15] Charlson ME Pompei P Ales KL MacKenzie CR A new method of classifying prognostic comorbidity in longitudinal studies: Development and validation J Chron Dis 1987 40 373 383 10.1016/0021-9681(87)90171-8 3558716

[B16] Clinical and Laboratory Standards Institute Performance standards for antimicrobial susceptibility testing: nineteenth and twenty first informational supplements 2009 Wayne, PA: Clinical and Laboratory Standards Institute M100 S19 and 2011:M100-S21

[B17] Pitout JD Gregson DB Campbell L Laupland KB Molecular characteristics of extended-spectrum-beta-lactamase-producing *Escherichia coli* isolates causing bacteremia in the Calgary Health Region from 2000 to 2007: emergence of clone ST131 as a cause of community-acquired infections Antimicrob Agents Chemother 2009 53 2846 2851 10.1128/AAC.00247-09 19380595 PMC2704701

[B18] Hunter SB Vauterin P Lambert-Fair MA Van Duyne MS Kubota K Graves L Wrigley D Barrett T Ribot E Establishment of a universal size standard strain for use with the PulseNet standardized pulsed-field gel electrophoresis protocols: converting the national databases to the new size standard J Clin Microbiol 2005 43 1045 1050 10.1128/JCM.43.3.1045-1050.2005 15750058 PMC1081233

[B19] Tenover FC Arbeit RD Goering RV Mickelsen PA Murray BE Persing DH Swaminathan B Interpreting chromosomal DNA restriction patterns produced by pulsed-field gel electrophoresis: criteria for bacterial strain typing J Clin Microbiol 1995 33 2233 2239 7494007 10.1128/jcm.33.9.2233-2239.1995 PMC228385

[B20] Heffernan HM Woodhouse RE Pope CE Blackmore TK Prevalence and types of extended-spectrum beta-lactamases among urinary Escherichia coli and Klebsiella spp. in New Zealand Int J Antimicrob Agents 2009 34 544 549 10.1016/j.ijantimicag.2009.07.014 19748232

[B21] Romero L Lopez L Rodreguez-bano J Ramon Hernandez J Martinez-Martinez L Pascual A Long term study of the frequency of *Escherichia coli* and *Klebsiella pneumoniae* isolates producing extended-spectrum beta-lactamases Clin Microbiol Infect 2005 11 625 631 10.1111/j.1469-0691.2005.01194.x 16008614

[B22] Marcade G Brisse S Bialek S Marcon E Leflon-Guibout V Passet V Moreau R Nicolas-Chanoine MH The emergence of multidrug-resistant *Klebsiella pneumoniae* of international clones ST13, ST16, ST35, ST48 and ST101 in a teaching hospital in the Paris region Epidemiol Infect 2013 141 1705 1712 10.1017/S0950268812002099 23034125 PMC9151615

[B23] Ma L Lu PL Siu LK Hsieh MH Molecular typing and resistance mechanisms of imipenem-non-susceptible *Klebsiella pneumoniae* in Taiwan: results from the Taiwan surveillance of antibiotic resistance (TSAR) study, 2002–2009 J Med Microbiol 2013 62 101 107 10.1099/jmm.0.050492-0 23002067

[B24] Freeman JT Williamson DA Heffernan H Smith M Bower JE Roberts SA Comparative epidemiology of CTX-M-14 and CTX-M-15 producing *Escherichia coli*: Association with distinct demographic groups in the community in New Zealand Eur J Clin Microbiol Infect Dis 2012 31 2057 2060 10.1007/s10096-011-1540-3 22271302

[B25] Nicolas Chanoine MH Jarlier V Robert J Arlet G Drieux L Leflon-Guibout V Laouénan C Larroque B Caro V Mentré F Group Coli β Patient’s origin and lifestyle associated with CTX-M-producing *Escherichia coli*: A case–control-control study PLoS One 2012 7 e30498 10.1371/journal.pone.0030498 22299043 PMC3267726

[B26] Harris AD Kotetishvili M Shurland S Johnson JA Morris JG Nemoy LL Johnson JK How important is patient to patient transmission in extended-spectrum β-lactamase *Escherichia coli* acquisition Am J Infect Control 2007 35 97 101 10.1016/j.ajic.2006.09.011 17327188

[B27] Harris AD Perencevich EN Johnson JK Paterson DL Morris JG Strauss SM Johnson JA Patient to patient transmission is important in extended-spectrum β-lactamase producing *Klebsiella pneumoniae* acquisition Clin Infect Dis 2007 45 1347 1350 10.1086/522657 17968833

[B28] Hilty M Betsch BY Bogli-Stuber K Heiniger N Stadler M Küffer M Kronenberg A Rohrer C Aebi S Endimiani A Droz S Mühlemann K Transmission dynamics of extended-spectrum β-lactamase-producing Enterobacteriaceae in the tertiary care hospital and the household setting Clin Infect Dis 2012 55 967 975 10.1093/cid/cis581 22718774 PMC3436924

[B29] Cholley P Thouverez M Gbaugidi-Haore H Sauget M Slekovec C Bertrand X Talon D Hocquet D Hospital cross-transmission of extended-spectrum β-lactamase *Escherichia coli* and *Klebsiella pneumoniae* Med Mal Infect 2013 43 331 336 10.1016/j.medmal.2013.06.001 23876202

[B30] Freeman JT Nimmo J Gregory E Tiong A De Almeida M McAuliffe GN Roberts SA Predictors of hospital surface contamination with extended-spectrum β-lactamase-producing Escherichia coli and Klebsiella pneumonia: patient and organism factors Antimicrob Resist Infect Control 2014 4 3 5 doi:10.1186/2047-2994-3-5 10.1186/2047-2994-3-5 PMC3922547 24491119

[B31] Moor CT Roberts SA Simmons G Briggs S Morris AJ Smith J Heffernan H Extended-spectrum beta-lactamase enterobacteria: factors associated with infection in the community setting, Auckland, New Zealand J Hosp Infect 2008 68 355 362 10.1016/j.jhin.2008.02.003 18353497

[B32] Rodriguez-Bano J Picon E Gijon P Hernández JR Ruíz M Peña C Almela M Almirante B Grill F Colomina J Giménez M Oliver A Horcajada JP Navarro G Coloma A Pascual A Spanish Network for Research in Infectious Diseases (REIPI) Community-Onset bacteraemia due to extended-spectrum beta-lactamase-producing *Escherichia coli*: Risk Factors and Prognosis Clin Infect Dis 2010 50 40 48 10.1086/649537 19995215

